# Comparable clinical outcomes of culture-negative and culture-positive periprosthetic joint infections: a systematic review and meta-analysis

**DOI:** 10.1186/s13018-023-03692-x

**Published:** 2023-03-16

**Authors:** Feng Li, Yongjie Qiao, Haoqiang Zhang, Guoding Cao, Shenghu Zhou

**Affiliations:** 1 Department of Joint Surgery, The 940th Hospital of Joint Logistic Support Force of Chinese People’s Liberation Army, Gansu, Lanzhou China; 2 Department of Orthopaedics, The 943rd Hospital of Joint Logistic Support Force of Chinese People’s Liberation Army, Gansu, Wuwei China; 3 Department of Orthopaedics, Lanzhou University Second Hospital, Gansu, Lanzhou China

**Keywords:** Periprosthetic joint infection, Culture negative, Outcome, Debridement, antibiotics and implant retention, Revision

## Abstract

**Purpose:**

The aim of this study was to compare the clinical outcomes of culture-negative periprosthetic joint infection (CN PJI) with those of culture-positive periprosthetic joint infection (CP PJI).

**Methods:**

Data were obtained from Embase, Web of Science and EBSCO for all available studies comparing the clinical outcomes of CN PJI with those of CP PJI. The quality of the studies was scored using the Newcastle–Ottawa scale (NOS). Pooled odds ratios (ORs) and 95% confidence intervals (CIs) were used to assess clinical outcomes. Subgroup analyses were performed to explain heterogeneity among the included studies. Publication bias was estimated using Begg’s funnel plot. Sensitivity analysis was performed to test the stability of pooled results.

**Results:**

Thirty studies with 1630 (38.7%) CN PJI and 2577 (61.3%) CP PJI were included in this meta-analysis. The pooled results of the included studies showed that overall failure rate in CN PJI group (19.0%, 309/1630) was significantly lower than that in CP PJI group (23.4%, 604/2577) (OR 0.63, 95% CI 0.47–0.84, *P* = 0.002). We performed the subgroup analysis based on the surgical strategies, the pooled results of nine studies for patients undergoing debridement, antibiotics and implant retention (DAIR) revealed that failure rate in CN PJI group (22.2%, 53/239) was significantly lower than that in CP PJI group (29.3%, 227/775) (OR 0.62, 95% CI 0.43–0.90, *P* = 0.01), the pooled results of four studies for patients undergoing one-stage revision revealed that failure rate between CN PJI group (11.5%, 11/96) and CP PJI group (7.6%, 27/355) had no significant difference (OR 1.57, 95% CI 0.75–3.26, *P* = 0.23), and the pooled results of 19 studies for patients undergoing two-stage revision revealed that failure rate in CN PJI group (16.1%, 171/1062) was significantly lower than that in CP PJI group (20.4%, 206/1010) (OR 0.52, 95% CI 0.34–0.79, *P* = 0.002).

**Conclusions:**

CN PJI group had similar or better survival rate when compared with CP PJI group for patients who underwent DAIR, one-stage or two-stage revision. Negative culture was not a worse prognostic factor for PJI.

**Supplementary Information:**

The online version contains supplementary material available at 10.1186/s13018-023-03692-x.

## Introduction

Periprosthetic joint infection (PJI) is a catastrophic complication after total joint arthroplasty (TJA), with incidence of approximately 1% [1] and 2% [2] after total hip arthroplasty (THA) and total knee arthroplasty (TKA), respectively. Surgical treatment options of PJI include debridement, antibiotics and implant retention (DAIR), one-stage or two-stage revision, arthrodesis and amputation [3]. The prevention, detection and treatment of PJI following TJA remains great challenge [4], particularly when cultures are negative. Culture-negative periprosthetic joint infection (CN PJI) was defined as the presence of purulence surrounding the prosthesis, a sinus tract communicating with the joint or positive histopathologic findings, in addition to there being no growth on aerobic and anaerobic cultures submitted to the clinical microbiology laboratory [5]. It is difficult to deliver targeted and effective antibiotic treatment of CN PJI due to lack of microbiological evidence. The incidence rate of CN PJI ranged from 7 to 42% with a pooled result of 11% in a systematic review [6]. In recent years, there have been numerous studies comparing the clinical outcomes of CN PJI and culture-positive PJI (CP PJI) treated with DAIR, one-stage revision and two-stage revision, but the conclusions are controversial. van Eck et al. [7] reported that the failure rate in CN PJI group was significantly lower than that in CP PJI group. Mortazavi et al. [8] reported that the failure rate in CN PJI group was significantly higher than that in CP PJI group. Xu et al. [9] and Mulpur et al. [10] reported the success rate of treatment for the CN PJI group was similar to that for the CP PJI group. The present study aims to give an overview on the current database of studies concerning CN PJI and evaluate whether CN PJI has a better or worse clinical outcomes when compared with CP PJI.

## Materials and methods

### Data and literature sources

This systematic review and meta-analysis adhered to the Preferred Reporting Items for Systematic reviews and Meta-Analyses (PRISMA) guidelines [11]. We performed a systematic search of various electronic databases (i.e., Embase, Web of Science and EBSCO) in November 2022 with the following search term: (total joint arthroplasty OR TJA OR total joint replacement OR TJR OR total knee arthroplasty OR TKA OR total knee replacement OR TKR OR total hip arthroplasty OR THA OR total hip replacement OR THR) AND (infection OR infections OR infected OR “periprosthetic joint infection” OR “prosthetic joint infection” OR PJI) AND (single-stage OR one-stage OR two-stage OR 2-stage OR revision OR revisions OR “irrigation and debridement” OR “I&D” OR “debridement, antibiotics, and implant retention” OR DAIR) AND (culture negative OR negative) AND (culture positive OR positive). All obtained by searching titles and abstracts were carefully evaluated, and then, full texts were screened to determine the included articles.

### Study selection

#### Inclusion and exclusion criteria

Two authors independently selected titles and abstracts as well as full-text articles from the above listed databases using the aforementioned search strategies, and a third author adjudicated discrepancies.

#### The inclusion criteria were listed as follows

(1) Retrospective or prospective studies comparing clinical outcomes of CN PJI versus CP PJI were included; (2) at least one of the following outcome measures was reported: success rate, failure rate, survival rate, infection control rate or reinfection rate; (3) without restrictions on age and sex were imposed; and (4) without limitations on race were imposed.

#### The following exclusion criteria were used

(1) Non-peer reviewed publications; (2) certain study designs (non-human trials, observational studies, case reports, case series, review articles and letters to the editor); (3) the inclusion and exclusion criteria for the study were not clear or reasonable; and (4) the full text cannot be obtained or the original data are incomplete.

#### Data extraction and quality assessment

The following data were extracted: 1) demographic and clinical information of the studies (including first author, year of publication, country, study type, study period, follow-up period, diagnostic criteria of PJI, sample size of CN PJI and CP PJI, joint involved, surgical strategies and antibiotic regimen); 2) outcome measures including success rate, failure rate, infection control rate or reinfection rate. Pertinent data were extracted by two reviewers independently from all eligible studies, and any disagreement was resolved by a third reviewer. Using the prior Delphi-based definition of success after treatment PJI [12], failure was defined as (1) failed infection eradication, characterized by a wound with fistula, drainage or pain, and reinfection by the same organism strain; (2) subsequent surgical intervention for infection after reimplantation surgery; or (3) occurrence of PJI-related mortality. Definitions of term used are illustrated in Additional file 2.

For each included study, the methodological quality was evaluated using Newcastle–Ottawa scale (NOS) [13] by two independent reviewers. The scores of each study were consisted of eight items with full mark of 9 scores. The studies with more than 6 scores were considered as high-quality article in our meta-analysis.

#### Statistical analysis

All analyses were conducted using the Review Manager software (Review Manager version 5.3, The Nordic Cochrane Centre, The Cochrane Collaboration 2014, Copenhagen, Denmark) and STATA software (STATA version 12.0). The Mantel–Haenszel model and odds ratios (ORs) with 95% confidence intervals (CIs) for outcomes of interest were used to compare dichotomous variables. A *P*-value less than 0.05 was considered statistically significant. We calculated the *I*^2^ coefficient to assess heterogeneity with the following predetermined limits: low < 50%, moderate 50–74% and high > 75%, and *P* ≥ 0.05 and *I*^2^ < 50% indicating no statistical heterogeneity between studies. A random-effects model was applied in circumstances of moderate or high heterogeneity; otherwise, a fixed-effects model was employed. If there was significant heterogeneity in the included studies, subgroup analysis was performed to explain heterogeneity. The Begg's funnel plots were used to evaluate publication bias. We judged that there was no publication bias if *P*-value was more than 0.05 for Begg’s test. Sensitivity analysis was performed to assess the stability of pooled results.

## Results

### Search strategy results

**Fig. 1:**
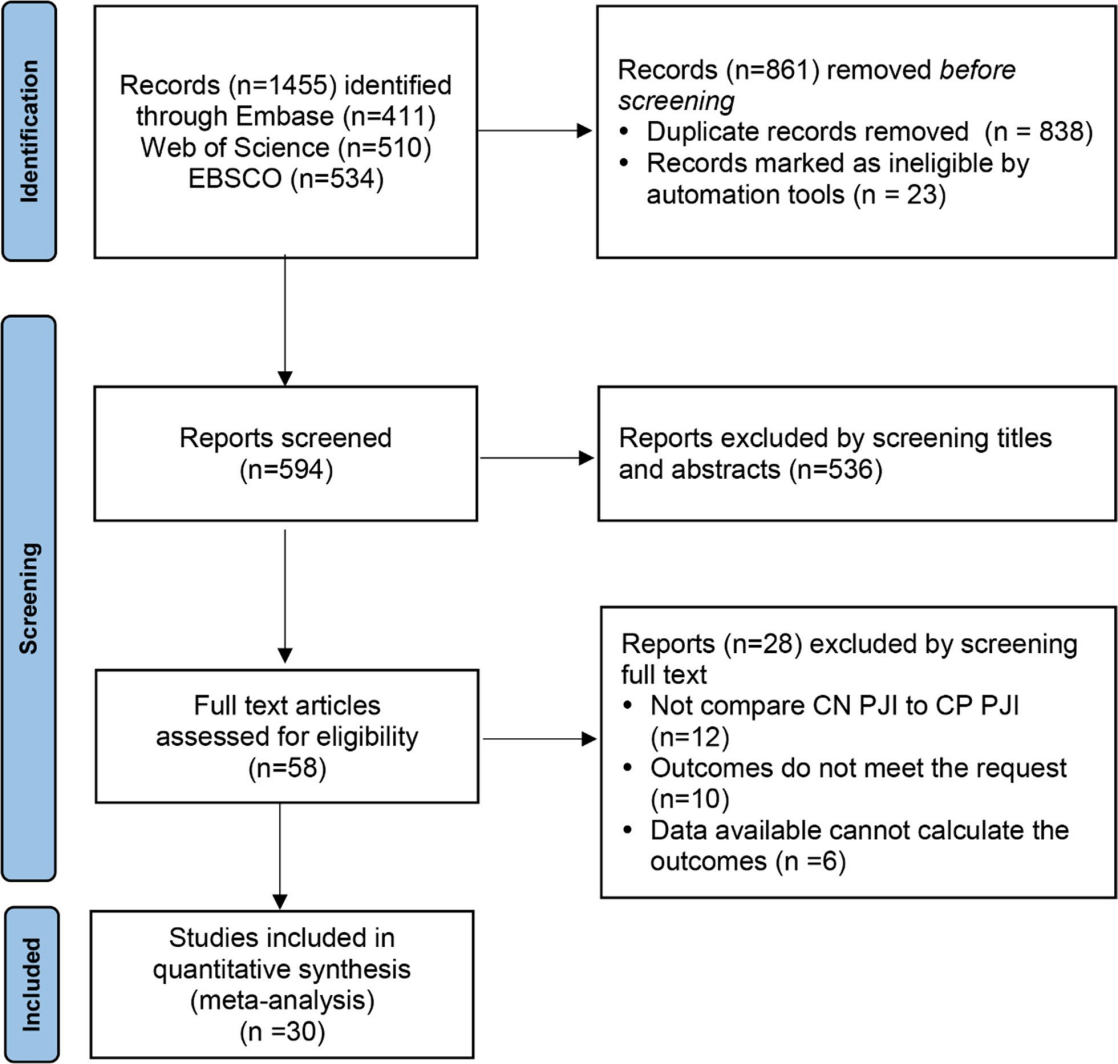
PRISMA flow diagram of the identification and selection of the studies included in this meta-analysis. EBSCO E.B.Stephens Company, *CN PJI* culture-negative periprosthetic joint infection, *CP PJI* culture-positive periprosthetic joint infection, *PRISMA* Preferred Reporting Items for Systematic reviews and Meta-Analyses

The search strategy previously described produced 1455 results (411 in Embase, 510 in Web of Science and 534 in EBSCO). Eight hundred and thirty-eight duplicates were excluded. After being reviewed the titles and abstracts by two independent authors, 536 irrelevant citations were removed. Subsequently, we assessed the remaining 58 full-text articles and excluded 28 articles based on the inclusion and exclusion criteria. Finally, 30 studies were included in our study and could be quantitatively synthesized and the remaining two were qualitatively analyzed. The article selection process is illustrated in Fig. 1.

### Study characteristics and quality assessment

In total, 28 retrospective studies [7–10, 14–37] and 2 prospective studies [38, 39] from 12 countries containing 4207 PJI cases were included in this meta-analysis. All studies were published between 2010 and 2022. Follow-up period ranged from 12 to 120 months. The diagnosis of PJI was based on the Musculoskeletal Infection Society (MSIS) criteria [40] in 22 studies, International Consensus Meeting (ICM) criteria [41] in 3 studies, Infectious Diseases Society of America (IDSA) [42] criteria in 1 study, Swiss Society of Infectious Diseases (SSID) criteria [27] in 1 study, and diagnostic criteria not acquire in 3 studies. The surgical strategies include DAIR, one-stage or two-stage revision and others. Furthermore, we assessed the quality of each included study and the NOS scores ranged from 6 to 9, which suggests the included studies are in a high quality. The main characteristics and quality assessment results of the included studies are given in Table 1.

**Table 1:** The main characteristics and quality assessment results of the included studies

Author	Year	Country	Study type	Study period	Follow-up (months)	Diagnostic criteria	Sample size	Joint involved		Surgical strategies				NOS scores
							CN PJI/CP PJI	Hip	Knee	DAIR	One-stage	Two-stage	Others	
van Eck [7]	2022	Netherlands	Retro	2014–2019	Minimum 12	ICM	60/299	228	131	359	0	0	0	8
Veerman [14]	2022	Netherlands	Retro	2012–2019	Minimum 24	IDSA	32/56	53	35	88	0	0	0	7
Xu [9]	2022	China	Retro	2012–2017	Mean 29.2	MSIS	22/48	n/a	n/a	21	13	36	0	8
Watanabe [16]	2021	Japan	Retro	2013–2019	Mean 51.5	ICM	27/82	79	24	21	10	66	12	9
Tirumala [17]	2021	USA	Retro	n/a	Minimum 36	ICM	46/103	59	90	149	0	0	0	7
Mulpur [10]	2021	India	Retro	2012–2018	Minimum 12	MSIS	27/53	0	80	80	0	0	0	8
Razii [15]	2021	UK	Retro	2006–2016	Mean 84	MSIS	16/68	84	0	0	84	0	0	6
Theil [18]	2020	Germany	Retro	2012–2016	Median 42	MSIS	153/51	93	111	0	0	204	0	8
Bongers [19]	2020	Netherlands	Retro	2003–2013	Minimum 24	MSIS	65/48	0	113	0	0	113	0	7
Ji [20]	2020	China	Retro	2009–2016	Mean 53.2	MSIS	51/192	104	139	0	243	0	0	8
Ji [21]	2019	China	Retro	2010–2016	Mean 58	MSIS	23/88	111	0	0	111	0	0	7
Xu [22]	2019	China	Retro	2012–2016	Minimum 12	MSIS	94/23	117	0	0	0	117	0	8
Wang [23]	2018	China	Retro	2003–2016	Median 68.5	MSIS	19/39	58	0	0	0	58	0	9
Kang [24]	2018	Korea	Retro	1996–2015	Mean 88.8	MSIS	15/70	85	0	0	0	85	0	8
Ibrahim [25]	2018	UK	Retro	2007–2012	Minimum 60	MSIS	50/50	100	0	0	0	100	0	9
Santoso [26]	2018	Indonesia	Retro	2010–2015	Mean 30.5	MSIS	27/57	84	0	0	0	84	0	9
Akgun [27]	2017	Germany	Retro	2013–2015	Mean 32.9	SSID	136/27	84	79	0	0	163	0	8
Li [28]	2017	China	Retro	2003–2014	Mean 59.5	MSIS	18/109	0	127	0	22	105	0	9
Bereza [39]	2016	Poland	Pro	n/a	Mean 32	MSIS	6/7	4	9	0	0	13	0	6
Tan [29]	2016	USA	Retro	1999–2013	Minimum 12	MSIS	234/33	81	186	0	0	267	0	9
Cha [30]	2015	Korea	Retro	1998–2011	Mean 30	MSIS	22/54	0	76	0	0	76	0	7
Triantafyllo-poulos [33]	2015	USA	Retro	2000–2013	Mean 79.7	MSIS	9/69	0	78	78	0	0	0	8
Kim [31]	2015	Korea	Retro	1991–2008	Mean 115.8	MSIS	51/140	0	191	109	0	82	0	9
Kim [32]	2015	Korea	Retro	2001–2008	Mean 120	MSIS	102/140	0	242	137	0	105	0	9
Nelson [34]	2014	USA	Retro	2010–2011	Mean 29.9	MSIS	18/18	7	29	0	0	36	0	6
Choi [35]	2013	USA	Retro	2000–2009	Mean 58	MSIS	40/135	78	97	54	0	110	11	9
Huang [36]	2012	USA	Retro	2000–2007	Mean 47	MSIS	48/295	n/a	n/a	97	5	238	3	9
Sorlí [38]	2012	Spain	Pro	2007–2008	Mean 12	n/a	44/11	17	37	0	0	55	0	6
Mortazavi [8]	2011	USA	Retro	1997–2007	Mean 45.6	n/a	40/77	0	117	0	0	117	0	6
Malekzadeh [37]	2010	Austria	Retro	1985–2000	Median 56	n/a	135/135	136	134	33	18	123	96	7

*CN PJI* culture-negative periprosthetic joint infection, *CP PJI* culture-positive periprosthetic joint infection, *DAIR* debridement, antibiotics and implant retention, *NOS* Newcastle–Ottawa scale, *MSIS* Musculoskeletal Infection Society, *ICM* International Consensus Meeting, *IDSA* Infectious Diseases Society of America, *SSID* Swiss Society of Infectious Diseases, n/a not acquire. *retro* retrospective, *pro* prospective

### Meta-analysis for overall failure rate

**Fig. 2:**
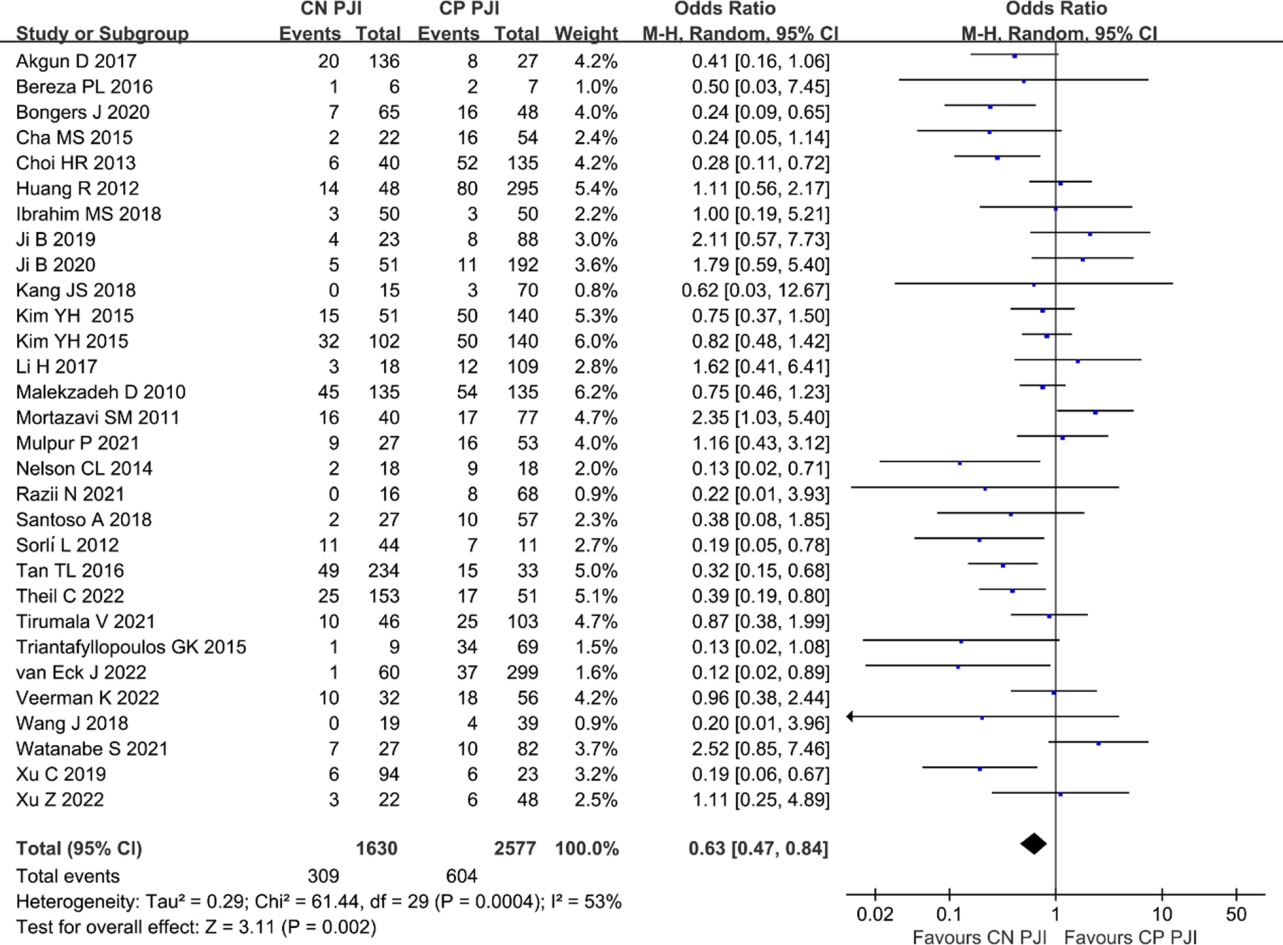
Forest plot showing the overall failure rate in patients with CN PJI versus with CP PJI. *CN PJI* culture-negative periprosthetic joint infection, *CP PJI* culture-positive periprosthetic joint infection

The overall treatment failure rate was 21.7% (913/4207) with a failure rate of 19.0% (309/1630) and 23.4% (604/2577) for CN PJI and CP PJI, respectively. Since there was moderate heterogeneity among all included studies [7–10, 14–39] (*I*^2^ = 53%, *P* = 0.0004), we performed a random-effects model to pool OR and 95% CI. As shown in Fig. 2, the pooled results also showed a lower treatment failure rate among patients with negative culture than those with positive culture (OR 0.63, 95% CI 0.47–0.84, *P* = 0.002).

### Subgroup analysis

**Fig. 3:**
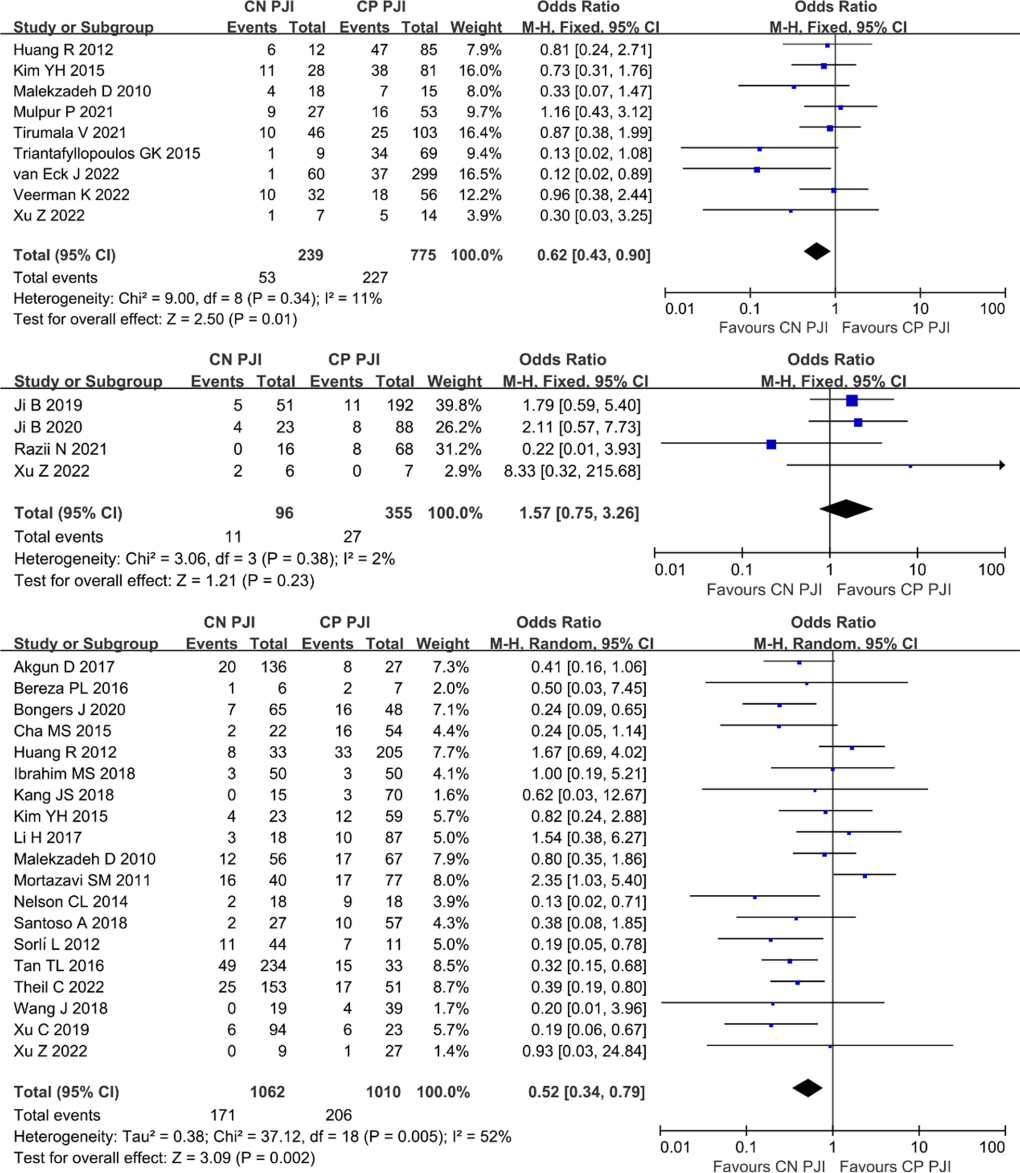
Forest plot showing the failure rate of subgroup analysis based on surgical strategies in patients with CN PJI versus with CP PJI. **A** DAIR, **B** one-stage revision, **C** two-stage revision. *CN PJI* culture-negative periprosthetic joint infection, *CP PJI* culture-positive periprosthetic joint infection, *DAIR* debridement, antibiotics and implant retention

Because moderate heterogeneity exists in overall failure rate results, subgroup analyses were performed to estimate the failure rates based on different surgical strategies. In the nine included studies [7, 9, 10, 14, 17, 31, 33, 36, 37] for patients who underwent DAIR, a fixed-effects model was performed due to no significant heterogeneity (*I*^2^ = 11%, *P* = 0.34). The pooled results revealed that CN PJI had a lower treatment failure rate than CP PJI (22.2% (53/239) vs 29.3% (227/775), OR 0.62, 95% CI 0.43–0.90, *P* = 0.01; Fig. 3A). In the four included studies [9, 15, 20, 21] for patients who underwent one-stage revision, a fixed-effects model was performed due to no significant heterogeneity (*I*^2^ = 2%, *P* = 0.38). The pooled results showed a similar treatment failure rate between CN PJI and CP PJI (11.5% (11/96) vs 7.6% (27/355), OR 1.57, 95% CI 0.75–3.26, *P* = 0.23; Fig. 3B). In the 19 included studies [8, 9, 18, 19, 22–31, 34, 36–39] for patients who underwent two-stage revision, a random-effects model was performed due to moderate significant heterogeneity (*I*^2^ = 52%, *P* = 0.005), The pooled results revealed that CN PJI had a lower treatment failure rate than CP PJI (16.1% (171/1062) vs 20.4% (206/1010), OR 0.52, 95% CI 0.34–0.79, *P* = 0.002; Fig. 3C).

### Publication bias and sensitivity analysis

**Fig. 4:**
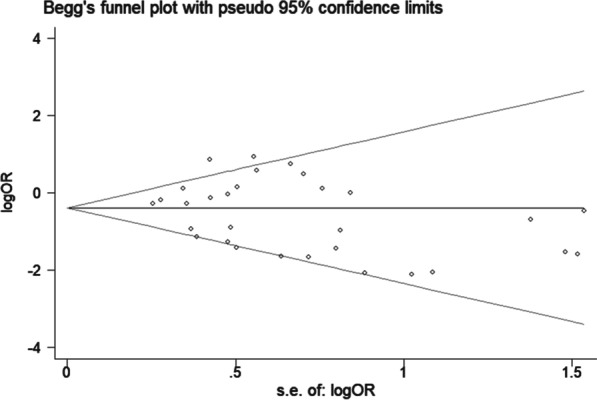
Begg’s funnel plot showed no significant publication bias among the included studies. *OR* odds ratios

As shown in Fig. 4, there was no obvious asymmetry in the Begg’s funnel plot, and the P value for Begg’s test was 0.318, which was greater than 0.05. Thus, there was no significant publication bias among the included studies.

**Fig. 5:**
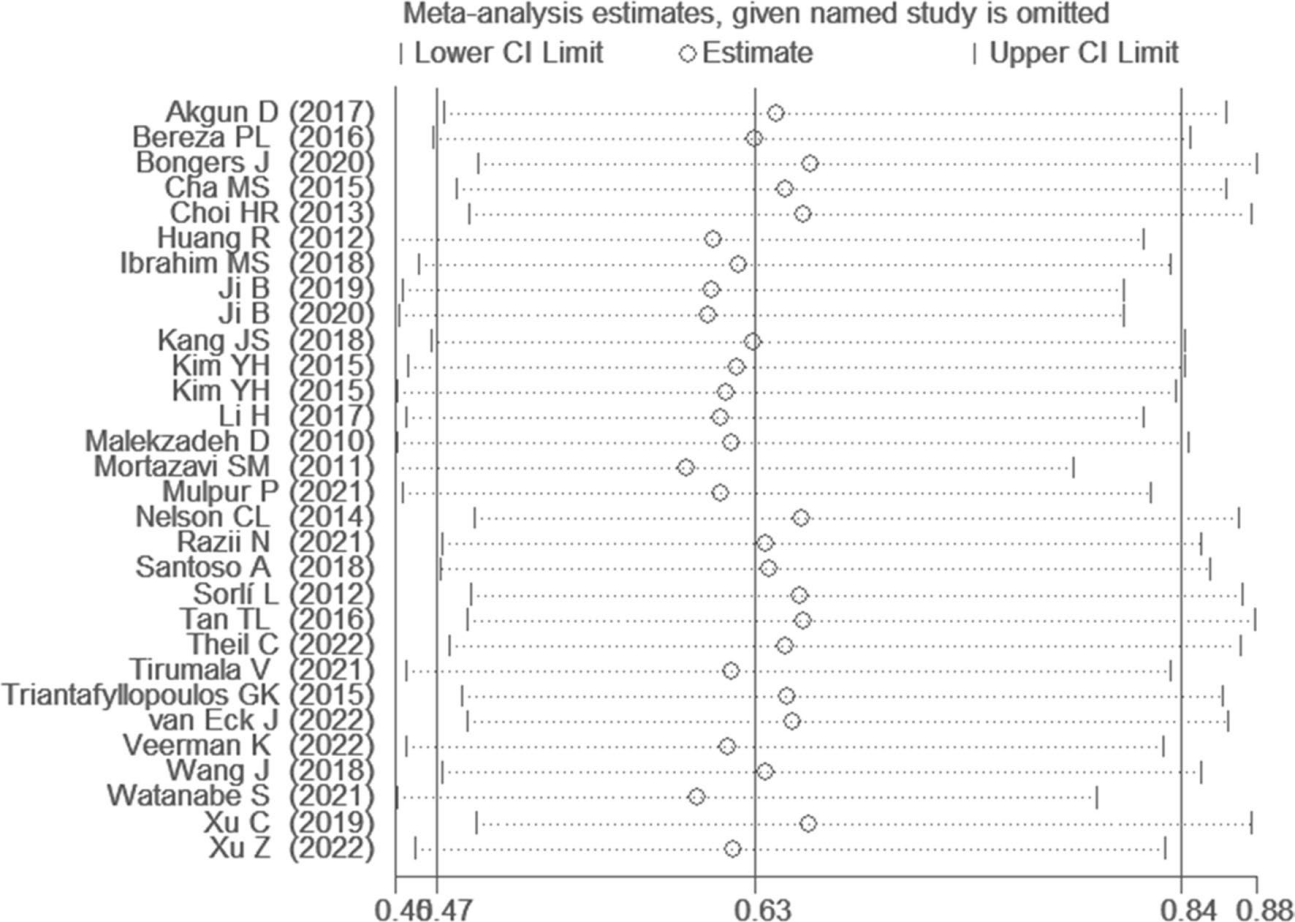
Sensitivity analysis showed no significant changes when each of the studies included were removed sequentially

Sensitivity analysis was applied to test the stability of pooled results. As shown in Fig. 5, the sensitivity analysis showed no significant changes when each of the studies included was removed sequentially.

## Discussion

The diagnosis, risk factors, treatment options and clinical outcomes of PJI have been widely discussed in the past two decades. However, data on CN PJI are relatively infrequent in the literature. Our study reported the failure rates in 1630 CN PJI cases and 2577 CP PJI cases who completed DAIR, one-stage or two-stage revision. We systematically collected relevant clinical trials of patients with CN PJI and CP PJI who underwent DAIR, one-stage and two-stage arthroplasty and then performed a meta-analysis and systematic review in this study. In this review of 30 studies including 4207 joints, we found that compared the outcomes of CN PJI with those of CP PJI after DAIR, one-stage or two-stage revision, it is suggested that negative culture may not be a negative prognostic factor for PJI. In contrast, we concluded that CN PJI patients have the better outcomes than CP PJI patients who underwent DAIR and two-stage arthroplasty, and patients undergoing a one-stage revision in the case of acute CN PJI have the same results compared to acute CP PJI.

### Treatment results of CN PJI and CP PJI patients following DAIR procedures

To date, the value of culture results after DAIR for acute PJI as risk indicators in terms of prosthesis retention remains controversial, and there is a paucity of data comparing the outcomes of DAIR between acute CN PJI and acute CP PJI. The results of this study are in accordance with those of van Eck et al. [7] and Malekzadeh et al. [37], we found the reinfection rate of CN PJI patients was lower than that of CP PJI patients after DAIR procedures (OR 0.62, 95% CI 0.43–0.90, *P* = 0.01), suggesting that negative culture may not be a contraindication to DAIR in patients with acute PJI. Kim et al. [31] retrospectively reviewed 140 patients with CP PJI and 102 patients with CN PJI also proved that controlled infection and maintained functional TKA with a firm level of fixation for most patients in both CP PJI and CN PJI groups, even repeated debridement also improved infection control rate after the initial treatment and increased the likelihood of maintaining a functional TKA. Similarly, a systematic review and meta-analysis concluded that CN PJI has the same or even better results than CP PJI including eight studies [6]. Both the aforementioned studies and our results showed that patients with CN PJI after DAIR procedure had the same or lower reinfection rate compared with patients with CP PJI. These results may be caused by low-virulence microorganisms, more thorough debridement during surgery, more strict perioperative management and longer antibiotic use. Furthermore, previous studies assessing DAIR procedures have shown the success rate is influenced by comorbidity, symptomatology, type of microorganism and especially timing of the DAIR procedure [33, 43–45]. The success rate extremely depended on the time frame between the surgical intervention and the start of symptoms. Löwik et al. [44] recommended that DAIR is a feasible option in patients with early PJI presenting more than 4 weeks after surgery, as long as DAIR is performed within at least 1 week after the onset of symptoms and modular components can be exchanged. Similarly, a retrospective study published by Shao et al. [46] reported success rate was 67.3% at a median 38.6 months follow-up in patients who underwent early surgery within ten days of the presentation of symptoms. Furthermore, there is a possibility that a negative culture could be the result of suboptimal diagnostic properties of cultures and was never infected in the first place, which may be one reason for the higher success rate. Another factor contributing to the high failure rate of CP PJI is the over-reliance on antibiotics by surgeons, which can result in the development of bacterial resistance. Therefore, extensive review of the local microbiological data used a multidisciplinary approach to optimize treatment protocols and improve the outcome for CP PJI patients. In conclusion, DAIR provided surgeons the possibility of curing the both acute CN PJI and CP PJI patients in appropriate time for surgery using a standard protocol during surgery and postoperatively can result in better outcomes, while at the same time retaining the implants, because it is thought to be associated with lower morbidity, less tissue fibrosis and better functional outcomes compared to the more invasive option of two-stage revision.

### Treatment results of CN PJI and CP PJI patients following one-stage arthroplasty

Of the previous studies, only a few recommended one-stage arthroplasty as the first treatment option. When the microorganism is determined, treatment results are well recorded in the literature. However, treatment results of CN PJI are only reported in a few studies. Although two-stage arthroplasty has traditionally been considered the gold standard of treatment for PJI, growing evidence is emerging in support of one-stage arthroplasty for selected patients. Our present study revealed that there was no significant difference in the success rate between the CN PJI group and the CP PJI group during one-stage revision (OR 1.57, 95% CI 0.75–3.26, *P* = 0.23). By the earliest one-stage arthroplasty procedure performed by von Foerster et al. [47], 76 cases were cured as a result of this single operation among 104 patients at follow-up period of 5–15 years. Buechel et al. [48] treated 22 infected TKAs by one-stage revision and followed for an average of 10.2 years, which found that the success rate achieved 90.9%. A retrospective study reported 70 patients who underwent one-stage arthroplasty with a rotating hinge with a minimum 9-year follow-up, which revealed that the infection-free survival was 93% [49]. However, the above-mentioned studies included positive culture only and used antibiotic-loaded polymethylmethacrylate (PMMA) cement in each case, which would lead to poor joint function, and the bias possibly affects the results. In recent years, one-stage arthroplasty treatment of CN PJI and CP PJI has gradually appealed to the surgeon's interest and achieved good results. Ji et al. [21] reported that 111 patients underwent routine one-stage revision with cementless reconstruction with powdered vancomycin or imipenem poured into the medullary cavity and reimplantation of cementless components at a mean follow-up time of 58 months; a recurrent infection was observed in four of the 23 patients (17.4%) with culture-negative infected hip. Not long after, the same medical center retrospectively analyzed 51 patients with CN PJI who underwent one-stage revision using intravenous and intra-articular antibiotic infusion compared with 192 patients with CP PJI at a mean of 53.2 months of follow-up, no significant difference in the infection control rate was observed between CN PJI and CP PJI (90.2% (46/51) versus 94.3% (181/192); *P* = 0.297) [20]. In addition, van den Kieboom et al. [50] considered one-stage revision demonstrated similar outcomes including reinfection, re-revision and readmission rates for the treatment of CN PJI after TKA and THA compared to two-stage revision. As there have significant physical, psychological and economic impacts with PJI, there are obvious advantages to operating one-stage revision, including reduced costs, less mortality, less time in hospital, decreased morbidity and higher patient satisfaction. But the criteria for one-stage revision in PJI, in principle, have been very strict, which reflect the complexity of cases and the need for a profitable condition to implant a new prosthesis. Contraindications to one-stage arthroplasty included culture-negative, significant tissue compromise, significant bone loss, systemic sepsis, immunosuppression, reinfection, multi-resistant organisms, polymicrobial infection, extensor mechanism failure or if primary wound closure was unlikely to be achievable [51, 52]. Therefore, this technique is still not widely used throughout the world due to restrictive inclusion criteria. The primary factor in the treatment at revision for CN PJI, whether one-stage or two-stage arthroplasty, following a thorough debridement, is the adequate and reasonable use of antibiotics. The spectrum of pathogens in published reports is almost similar, hence, the use of antibiotics with the broadest possible range, which can combat both gram-negative and gram-positive organisms, even CN PJI will cover almost all microorganisms. Moreover, in order to get better or comparable outcomes for CN PJI patients, surgeons are more careful while performing debridement, employing medications in conjunction with vancomycin and imipenem or meropenem and prescribing antibiotics for longer durations compared to CP PJI patients. To sum up, the surgeon should control indications and contraindications strictly, one-stage revision can be effective in the treatment of CN PJI and can achieve an infection control rate similar to that in CP PJI. Nonetheless, the patients in CP PJI may require further medical optimization and prior to one-stage revision to enhance their immune system, and a standardized diagnostic protocol and evidence-based treatment strategies for CN PJI should be implemented for further studies.

### Treatment results of CN PJI and CP PJI patients following two-stage arthroplasty

Although two-stage arthroplasty is today considered as the gold standard for treating chronic PJI, the reported success rate is very variable, ranging from 64 to 100% [8, 9, 53–55]. In addition, CN PJI will complicate diagnosis and management of PJI, and a lack of identification of an infecting organism preoperatively is an unfavorable factor of reimplantation. However, our meta-analysis demonstrated that negative culture at two-stage reimplantation instead of increasing the risk for reinfection greatly improved the success rate compared with positive culture (OR 0.52, 95% CI 0.34–0.79, *P* = 0.002). In agreement with our results, most of the previous studies concluded that CN PJI had the same or even better results than culture-positive infections. Choi et al. [35] retrospectively reviewed 40 culture-negative patients and 135 culture-positive patients demonstrating that the success rate of infection control was higher in the culture-negative group (*P* = 0.006) undergoing two-stage reimplantation. Another retrospective cohort study also showed that data from 77 patients who underwent two-stage revision to PJI after hip and knee arthroplasty were followed regularly with an average of 29.2 months; the infection control rate for the CN PJI group was similar to that for the CP PJI group [9]. On the contrary, Mortazavi et al. [8] identified a prospective database contained 117 patients who underwent two-stage arthroplasty, the multivariate analysis provided culture-negative (OR 4.5; 95% CI 1.3–15.7), methicillin-resistant organisms (OR 2.8; 95% CI 0.8–10.3) and increased reimplantation operative time (OR 1.01; 95% CI 1.0–1.03) as predictors of failure, and CN PJI increases the risk of failure over fourfold; however, this study was early and the bacterial culture technique was poor, so many positives may not have been cultured. There are many other studies that show that CN PJI has the better infection control rate, but there was no significant difference in the success rate between the CN PJI group and the CP PJI group during two-stage revision [18, 23, 25, 36]; this may explain the superior cure rate of CN PJI in the pooled results. Many factors may influence the outcomes of two-stage arthroplasties theoretically, including timing of reimplantation, serum markers, history of surgeries, the patient’s comorbidities, medical conditions, bone stock, soft tissue integrity and organism virulence; patients with these conditions are poor hosts and may thus be vulnerable to a new infection. Determining the appropriate timing of when reimplantation should be performed is often challenging for the treating surgeon. Khury et al. [56] and Stambough et al. [57] indicated that no association could be determined between the delta change in serum WBC, CRP and ESR before and after two-stage revision for PJI and reinfection risk, although a return to normal serology infrequently occurs before reimplantation, and Ackmann et al. [58] considered plasma D-dimer does not help to guide the timing of reimplantation in two-stage exchange for PJI; these serum markers provide no additional diagnostic accuracy to determine the timing of reimplantation. Another retrospective study by Fu et al. [59] proved that the proper timing of reimplantation should be combined with disappearance of clinical symptoms and negative intraoperative frozen section with spacer detention time at 12 to 16 weeks. As far as we know, the optimal timing of when reimplantation in two-stage revision remains unknown, so further studies are needed to resolve these questions. Furthermore, PJI with biofilm-forming organisms is a leading cause of failure and reinfection after two-stage reimplantation [34], because it is often difficult to detect such infections, particularly in patients who have received antibiotic treatment before surgery. Finally, methicillin-resistant or high-virulence microorganisms are positive culture, more comorbidities and increased reimplantation operative time as predictors of failure and reinfection after two-stage reimplantation [8, 27, 60]. In conclusion, our present study revealed that better results were obtained with negative culture than with positive culture. Therefore, appropriate timing of surgery, well-managed comorbidities, thorough debridement and effective antibiotic use are all beneficial to success rate and the CN PJI is not contraindications of two-stage revision.

Moreover, there are several possible reasons that an infective organism might not be confirmed preoperatively, including pre-operative use of antibiotics, an insufficient period without antibiotics before sampling, inadequate culture times or culture medium, low-virulence organisms, bacterial biofilms, limitations of sampling techniques or the lack of diagnostic facilities for rare organisms [7, 20]. As PJI is frequently caused by low-virulence organisms that might require prolonged incubation periods, to increase the detection rate of the low-virulence microorganisms multiple samples (minimum 3) should be taken, and an adequate growth time of at least 14 days [14, 38]. Sonication of explanted components is a new and more sensitive method for diagnosing infection that has proved to be effective, particularly in patients who had received antimicrobial treatment within 14 days before surgery [34, 61]. Sonication has been also reported to be a reliable tool for the diagnosis of an infected arthroplasty and subsequent biofilm-related infections [38], and it is crucial for the second-stage arthroplasty because spacers can act as a foreign body on to which bacteria may adhere [34]. In addition, arthroscopic sampling and polymerase chain reaction are necessary, as these patients were considered as having a complex CN PJI [62, 63]. Even though recent most studies concluded that CN PJI has the same or even better results than CP PJI following DAIR, one-stage and two-stage revision, selection of antibiotics is challenging in the absence of information about the causative organism. Empirical antibiotic use for CN PJI patients who underwent DAIR, one-stage and two-stage revision was comparable to antibiotic use in CP PJI patients according to a reliable antimicrobial susceptibility test, but the duration of antibiotic medication may be longer, which will increase economic burden, drug toxicity, damage liver and kidney function and psychological impacts.

### Limitations

Some limitations must be taken into consideration when interpreting the results of this study. Firstly, most of the included studies in our meta-analysis were mainly retrospective case–control studies and cohort studies with limitations inherent to such a study design, with no randomized controlled trials studies, so more prospective studies and confounders controlled are warranted to evaluate the clinical outcomes of CN PJI and CP PJI patients. Secondly, there were no single standard on other potential confounders, such as length of surgery time, blood loss, follow-up time, duration of antibiotic use, antibiotic treatment regimen and other non-measurable factors (e.g., the types of implants, surgical technique, surgical approach, etc.). Further research is necessary to elucidate for these findings. Thirdly, the diagnostic criteria for PJI are different, the different definitions of reinfection or cure is a potential criticism of every study assessing PJI in some studies; due to the lack of advanced culture techniques, infections caused by slow-growing pathogens such as mycobacteria or fungi were classified as CN PJI in previous studies, such a high rate of misclassification may threaten our study and we cannot analyze these risk factors or outcomes in this study. Perhaps we might be able to solve this issue by increasing the diagnostic accuracy of CN PJI using next-generation sequencing or a special culture medium, both of which have shown to be highly accurate in CN PJI diagnosis in subsequent studies. Fourth, several of the included studies had an identical author with overlap study period, and we confirmed that there is also partial overlap of reported population. However, despite using strict inclusion and exclusion criteria, we were unable to eliminate the overlap population. The pooled results may be impacted. So, sensitivity analysis was used to test the stability of pooled results. However, the sensitivity analysis showed no significant changes when each of the studies included were removed sequentially. Fifth, the majority of the included studies reflect the survival rates of CN PJI and CP PJI in the short- and medium-term. To demonstrate that the one-stage revision of CN PJI and CP PJI can result in the same or a higher survival rate, more long-term follow-up studies are required; Finally, the included studies used a mixed cohort of hips and knees and we thus were unable to investigate the independent results for hips in our meta-analysis, the possibility of not having retrieved all relevant information published on CN PJI should also be considered as one of the limitations of our study. These recognized limitations are inherent to all studies using this database design and could potentially be improved through prospective data collection.

## Conclusions

To our knowledge, this is the first study that has compared the clinical outcomes of CN PJI and CP PJI patients who underwent DAIR, one-stage or two-stage revision. Our study demonstrated that CN PJI patients had the better survival rate compared to CP PJI patients who underwent DAIR and two-stage revision, and a one-stage revision procedure in the case of CN PJI had the similar survival rate compared to CP PJI. Although CN PJI patients remain challenging to make exact diagnosis, suitable treatment and choose appropriate antibiotics, as the through debridement was considered imperative in every case, DAIR, one-stage and two-stage revision arthroplasty suggested that negative culture was not a worse prognostic factor for PJI.

## Supplementary Information

**Additional file 1.** Availability of data and materials.

**Additional file 2: Table S1**: Definitions of terms used.

## Data Availability

All data generated or analyzed during this study are included in Additional file 1.

## Abbreviations

CN PJI: Culture-negative periprosthetic joint infection
CP PJI: Culture-positive periprosthetic joint infection
DAIR: Debridement, antibiotics and implant retention
NOS: Newcastle–Ottawa scale
MSIS: Musculoskeletal Infection Society
ICM: International Consensus Meeting
IDSA: Infectious Diseases Society of America
SSID: Swiss Society of Infectious Diseases
