# Supplementary material for: Comparable clinical outcomes of culture-negative and culture-positive periprosthetic joint infections: a systematic review and meta-analysis
Source: J Orthop Surg Res. 2023 Mar 16;18:210. doi: 10.1186/s13018-023-03692-x (PMC10018887; doi:10.1186/s13018-023-03692-x)
Supplement: Supplementary file 2 — Additional file 2: Table S1: Definitions of terms used. [file 13018_2023_3692_MOESM2_ESM.docx]

Supplementary table 1. Definitions of terms used

| **MSIS criteria** |
| --- |
| (1) There is a sinus tract communicating with the prosthesis; or  (2) A pathogen is isolated by culture from at least two separate tissue or fluid samples obtained from the affected prosthetic joint; or  (3) Four of the following six criteria exist:  (a) Elevated serum erythrocyte sedimentation rate (ESR) and serum C-reactive protein (CRP) concentration,  (b) Elevated synovial leukocyte count,  (c) Elevatedsynovialneutrophilpercentage(PMN%),  (d) Presence of purulence in the affected joint,  (e) Isolation of a microorganism in one culture of periprosthetic tissue or fluid, or  (f) Greater than five neutrophils per high-power field in five high-power fields observed from histologic analysis of periprosthetic tissue at 9400 magnification. |
| **ICM** **criteria** |
| Two positive periprosthetic cultures with phenotypically identical organisms, or A sinus tract communicating with the joint, or Having 3 of the following minor criteria:- Elevated serum; C-reactive protein (CRP) AND erythrocyte sedimentation rate (ESR); Elevated synovial fluid white blood cell (WBC) count; OR ++ change on leukocyte esterase test strip; Elevated synovial fluid polymorphonuclear neutrophil percentage (PMN%); Positive histological analysis of periprosthetic tissue; A single positive culture. |
| **IDSA criteria** |
| (1). The presence of a sinus tract that communicates with the prosthesis is definitive evidence of PJI.  (2). The presence of acute inflammation as seen on histopathologic examination of periprosthetic tissue at the time of surgical debridement or prosthesis removal as defined by the attending pathologist is highly suggestive evidence of PJI.  (3). The presence of purulence without another known etiology surrounding the prosthesis is definitive evidence of PJI.  (4). Two or more intraoperative cultures or combination of preoperative aspiration and intraoperative cultures that yield the same organism (indistinguishable based on common laboratory tests including genus and species identification or common antibiogram) may be considered definitive evidence of PJI. Growth of a virulent microorganism (eg, S. aureus) in a single specimen of a tissue biopsy or synovial fluid may also represent PJI. One of multiple tissue cultures or a single aspiration culture that yields an organism that is a common contaminant (eg, coagulase-negative staphylococci, Propionibacterium acnes) should not necessarily be considered evidence of definite PJI and should be evaluated in the context of other available evidence (B-III).  (5). The presence of PJI is possible even if the above criteria are not met; the clinician should use his/her clinical judgment to determine if this is the case after reviewing all the available preoperative and intraoperative information. |
| **SSID criteria** |
| Not acquire. |
| **Culture-negative PJI** |
| No growth of either aerobic or anaerobic cultures taken from periprosthetic tissue during surgery or by  periprosthetic aspiration from patients with THA or TKA with at least one of the following:  (1) Presence of periprosthetic purulence observed during surgery or at the time of aspiration  (2) The presence of acute inflammation on periprosthetic tissue surgical specimens by the histopathologist  (3) The presence of a cutaneous sinus tract communicating with the prosthesis |
| **Culture-positive PJI** |
| Isolation of the same microorganism from two or more cultures of joint aspirates or intraoperative tissue  specimens or the isolation of a single microorganism with at least one of the following:  (1) Presence of periprosthetic purulence observed during surgery or at the time of aspiration  (2) The presence of acute inflammation on periprosthetic tissue surgical specimens by the histopathologist  (3) The presence of a cutaneous sinus tract communicating with the prosthesis |
| **Treatment failure** |
| (1) failed infection eradication, characterized by a healed wound without fistula, drainage, or pain, and no reinfection by the same organism strain;  (2) subsequent surgical intervention for infection after reimplantation surgery; or  (3) occurrence of PJI-related mortality. |
